# Cystathionine β-Synthase Is Necessary for Axis Development *in Vivo*

**DOI:** 10.3389/fcell.2018.00014

**Published:** 2018-02-16

**Authors:** Shubhangi Prabhudesai, Chris Koceja, Anindya Dey, Shahram Eisa-Beygi, Noah R. Leigh, Resham Bhattacharya, Priyabrata Mukherjee, Ramani Ramchandran

**Affiliations:** ^1^Department of Pediatrics, Medical College of Wisconsin, Milwaukee, WI, United States; ^2^Department of Obstetrics and Gynecology, University of Oklahoma Health Science Center, Oklahoma City, OK, United States; ^3^Pediatrics Radiology, Developmental Vascular Biology Program, Children's Research Institute, Medical College of Wisconsin, Milwaukee, WI, United States; ^4^Milwaukee Health Department, City of Milwaukee, Milwaukee, WI, United States; ^5^Peggy and Charles Stephenson Cancer Center, University of Oklahoma Health Science Center, Oklahoma City, OK, United States; ^6^Department of Pathology, University of Oklahoma Health Science Center, Oklahoma City, OK, United States; ^7^Department of Cell Biology, University of Oklahoma Health Science Center, Oklahoma City, OK, United States; ^8^Obstetrics and Gynecology, Medical College of Wisconsin, Milwaukee, WI, United States

**Keywords:** zebrafish, CRISPR, small molecules, methionine, homcystinuria, hydrogen sulfide, morpholino

## Abstract

The cystathionine ß-synthase (CBS) is a critical enzyme in the transsulfuration pathway and is responsible for the synthesis of cystathionine from serine and homocysteine. Cystathionine is a precursor to amino acid cysteine. CBS is also responsible for generation of hydrogen sulfide (H_2_S) from cysteine. Mutation in *CBS* enzyme causes homocysteine levels to rise, and gives rise to a condition called hyperhomocysteinuria. To date, numerous mouse knockout models for CBS enzyme has been generated, which show panoply of defects, reflecting the importance of this enzyme in development. In zebrafish, we and others have identified two orthologs of *cbs*, which we call *cbsa* and *cbsb*. Previous gene knockdown studies in zebrafish have reported a function for *cbsb* ortholog in maintaining ion homeostasis in developing embryos. However, its role in maintaining H_2_S homeostasis in embryos is unknown. Here, we have performed RNA analysis in whole zebrafish embryos that showed a wide expression pattern for *cbsa* and *cbsb* primarily along the embryonic axis of the developing embryo. Loss-of-function analysis using a combination of approaches which include splice morpholinos and CRISPR/Cas9 genomic engineering show evidence that *cbsb* ortholog is responsible for anterior-posterior axis development, and *cbsa* function is redundant. *Cbsb* loss of function fish embryos show shortened and bent axis, along with less H_2_S and more homocysteine, effects resulting from loss of Cbsb. Using a chemical biology approach, we rescued the axis defects with betaine, a compound known to reduce homocysteine levels in plasma, and GYY4137, a long term H_2_S donor. These results collectively argue that cells along the axis of a developing embryo are sensitive to changes in homocysteine and H_2_S levels, pathways that are controlled by Cbsb, and thus is essential for development.

## Introduction

Cystathionine ß-synthase (CBS) and cystathionine γ-lyase (CSE) are two key enzymes that are involved in the biosynthesis of hydrogen sulfide (H_2_S), a key signaling molecule that participates in various physiological functions in vertebrates (Huang and Moore, 2015). Endogenous H_2_S biosynthesis is also catalyzed by the coordinated action of two other enzymes cysteine aminotransferase (CAT) and 3-mercaptopyruvate sulfur transferase (3-MST). In addition to H_2_S biosynthesis, CBS and CSE enzymes also participate in transsulfuration reactions involving cystathionine (precursor to cysteine) biosynthesis through a condensation reaction of serine and homocysteine (Jhee and Kruger, 2005). Hyperhomocysteinemia (HHcy), a condition with elevated homocysteine is associated with mutation in the CBS enzyme (Kraus et al., 1999). HHcy is classified based on levels of homocysteine that range from 10.8 to 12.4 mmol/L (normal), 15 to 30 mmol/L (moderate), 31 to 100 mmol/L (intermediate), or >100 mmol/L (severe) (Födinger et al., 1999). Neural tube defects resulting from failure of neural tube closure during the first 28 weeks of pregnancy remain the leading developmental disorder in these patients. Oral cleft, congenital heart defects and cardiovascular disorders were also observed in these patients. In adults, HHcy is associated with several conditions including diabetes, pulmonary embolism and Alzheimer's disease. Thus, CBS remains an important target that requires further understanding (Majtan et al., 2016).

The CBS pathway is a gateway to several essential biochemical processes including glutathione synthesis. Glutathione is a tripeptide that is synthesized from cysteine, and is vital for detoxification reaction in cells, and thus serves an anti-oxidant function. CBS generates key precursors such as cysteine, thus serving at a critical intersection point in pathways associated with endogenous detoxification mechanisms and biosynthesis of H_2_S in the cell. It is therefore not surprising that *Cbs* is critical for development and homozygous *Cbs* knockout in mice die *in utero* (Watanabe et al., 1995). Majority of published studies on *Cbs* knockout mice is performed on heterozygous *Cbs* background. Numerous defects ranging from vascular endothelial dysfunction (Lentz et al., 2000; Dayal et al., 2001), redox homeostasis (Vitvitsky et al., 2004), hyperkeratosis (Robert et al., 2004), endochondrial ossification (Robert et al., 2005), retinal neuron death (Ganapathy et al., 2009), lung fibrosis (Hamelet et al., 2007b), and hepatic steatosis (Hamelet et al., 2007a) have been reported. Majority of these defects have in part been attributed to elevated plasma homocysteine levels. A human *CBS* knock-in transgene in the endogenous mouse *Cbs* locus has also been generated, which shows elevations in plasma and tissue levels of homocysteine but shows mild hepatopathy and no hepatic steatosis or fibrosis in contrast to classical models of homcystinuria (MacLean et al., 2010). In lower species, such as zebrafish, gene knockdown of either *cbs* or *cse* using antisense morpholino oligonucleotides (MOs) (Porteus et al., 2014) influenced the hypoxic ventilatory response, an adaptation under stress conditions that allows organism to intake and process oxygen at higher rates. The oxygen-sensing neuroepithelial cells in zebrafish showed an increase in intracellular calcium concentration. The same group later reported that *cbsb* knockdown using morpholinos (MOs) and not *cse* showed a reduction in calcium influx in larval zebrafish (Kwong and Perry, 2015). In addition to calcium, larval zebrafish containing gene knockdowns of *cse* and *cbsb* (Kumai et al., 2015) showed better sodium uptake. Collectively, these results argue for a role for *cbsb* in maintaining H_2_S and homocysteine homeostasis in developing embryos. However, the consequence of altering H_2_S and homocysteine homeostasis via modulation of *cbsb* during zebrafish embryonic development is unknown. Here, we performed a systematic and detailed analysis into Cbs enzyme role in zebrafish embryonic development, and its role in maintaining H_2_S and homocysteine homeostasis *in vivo*. Because of genome duplication in zebrafish, a significant portion of the genes in zebrafish have duplicates (Postlethwait et al., 2000). For the *cbs* gene, of the two orthologs *cbsa* and *cbsb* in zebrafish, we postulated that the *cbsb* gene was critical for embryonic development because of prior *in situ* hybridization (ISH) pattern data observed on zebrafish information network site. To investigate this hypothesis, we performed RNA analysis and whole mount ISH (WISH) across embryonic stages. We also performed loss-of-function analysis using MOs (Ekker, 2000) and CRISPR/Cas9 (Hruscha et al., 2013)-based genomic engineering approaches. Finally, we performed rescue for the loss-of-function phenotypes using small molecules that modulate the H_2_S and cysteine biosynthesis pathway. Our study here points to a critical role for *cbsb* in embryonic axis development.

## Materials and methods

### Zebrafish

All zebrafish studies performed here were carried under the AUA protocol 320, which is approved by the MCW institutional animal care and use committee. Embryo stages were performed as per the zebrafish book and whole mount *in situ* hybridization with anti-sense and sense digoxigenin probes were carried out based on protocols published before (Bedell et al., 2005; Thisse and Thisse, 2014).

### Chemicals

GYY4137 (P-(4-Methoxyphenyl)-P-4-morpholinyl-phosphinodithioic acid), NaSH.xH_2_O (sodium hydrosulfide hydrate) and betaine (Trimethylglycine) chemicals were purchased from Sigma Aldrich. 1 M Stock solution of GYY4137 was prepared in dimethylsulfoxide (DMSO) and stored at 4°C. 50 mg/ml solution of betaine was prepared in water and stored at 4°C. 1 M stock solution of NaSH.xH_2_O was prepared in water and used immediately.

### Molecular biology

Probes for sense and anti-sense *cbsa* and *cbsb* were generated using PCR-based methods. The primers for these probes include:

*Cbsa* sense: Fwd 5′-CACCGAAATAATACGACTCACTATAGGGGATGGAGACAGACCCCCACA, Rev 5′-GGCACTTTTCCTTCAATTTCCGA and antisense primers: Fwd 5′- GATGGAGACAGACCCCCACA, Rev 5′ CACCGAAATTAACCCTCACTAAAGGGGGCACTTTTCCTTCAATTTCCGA

*Cbsb* sense: Fwd 5′-CACCGAAATAATACGACTCACTATAGGGATCAACGGGGATGCTGACGAT, Rev 5′- CAATCTCAGCACCCAGAGCA and antisense primers: Fwd 5′- ATCAACGGGGATGCTGACGAT, Rev 5′- CACCGAAATTAACCCTCACTAAAGGGCAATCTCAGCACCCAGAGCA

Primer sequences used for amplification include:

*Cbsa*, Fwd 5′- GATGGAGACAGACCCCCACA, Rev 5′- GGCACTTTTCCTTCAATTTCCGA

*Cbsb*, Fwd 5′- ATCAACGGGGATGCTGACGAT, Rev 5′- CAATCTCAGCACCCAGAGCA

Actin (*actb1*): Fwd 5′- GAAATTGTCCGTGACATCAA, Rev 5′- CACTGTGTTGGCATACAGGT

### Western blotting and IF

For western blotting, 48 h post fertilization (hpf) CBSB-S1-injected, ConMO-injected and *cbsb* CRISPR embryos were dechorionated and then de-yolked in PBS. Total cell lysate was prepared in radioimmunoprecipitation assay (RIPA) buffer containing protease inhibitors. Measurement of protein concentration was performed using BCA assay kit from Pierce, Grand Island, NY, USA. SDS-PAGE and immunoblotting was performed using standard protocol. The cell lysates were separated on 10% glycine SDS-PAGE gel and transferred to PVDF membrane. Membranes were blocked in 5% BSA in TBS with 0.1% TWEEN-20 (TBST) for 1 h at room temperature followed by incubation with indicated primary antibodies in TBST with 5% BSA. Antibodies were purchased from following vendors. CBS (D8F2P) purified rabbit antibody (catalog #14782, Cell Signaling Technology, MA) was used at a 1:1000 dilution; anti-GAPDH antibody (Sigma-Aldrich, MO) was used at a 1:5,000 dilution at 4°C overnight. Horseradish peroxidase conjugated secondary antibodies were used and proteins were visualized by Clarity Western ECL Substrate (Bio-Rad). For the immunofluorescence experiment, CBSB-S1-injected embryos, ConMO-injected embryos and *cbsb*-CRISPR embryos were dechorionated at 48 hpf and fixed with 4% paraformaldehyde in phosphate buffered saline (PBS) at 4°C overnight. The following day, embryos were washed twice with PBST (PBS, 0.1% (v/v) Tween 20), permeabilized in ice-cold acetone for 10 min and washed in PBST. Embryos were blocked in 10% normal goat serum for 1 h and incubated with purified rabbit anti-homocysteine antibody (Catalog # ab15154, Abcam, MA) overnight at 4°C. Embryos were washed twice with PBST, incubated with the appropriate secondary antibody (Alexa fluor 568 goat anti-rabbit, Invitrogen, MA) for 2 h at RT, washed with PBST and imaged on a Zeiss AxioObserver Z1 fluorescence inverted microscope.

### Morpholino studies

Gene Tools, Inc. designed all the morpholinos. Sequence is provided (**Figure 2C**). Control MO sequence used in this study is 5′-CCTCTTACCTCAGTTACAATTTATA-3′. All MOs were each injected into Transgenic (Tg: *flk*:EGFP) (Choi et al., 2007) one cell stage embryos. The 1 mM MO stock solutions were diluted to a final concentration of 2 ng/nL (250 μM), and appropriate concentrations as shown in the figure panels were injected into each embryo. For phenotypic imaging, 30 and 52 hpf embryos were anesthetized in 0.02% tricaine, mounted on a depression slide, and imaged with a Zeiss Stemi 2000-C dissecting microscope.

### H_2_S measurement studies

H_2_S was measured according to the published protocol (Papapetropoulos et al., 2009). Briefly, ConMO-injected and CBSB-S1-injected zebrafish embryos were collected by centrifugation and re-suspended in a total volume of 200 μL of PBS (pH 7.4), and 100 μL was then transferred directly into a tube containing zinc acetate (1% wt/vol, 187.5 μl) and NaOH (12%, 12.5 μL) to trap the H_2_S for 20 min at room temperature. The rest of the embryo suspension was used to estimate protein concentration by BCA assay. The reaction was terminated by adding 1 mL of H_2_O (pH 12.8), 200 μL of N, N-dimethyl-p-phenylenediamine sulfate (20 mM in 7.2 M HCl), and 200 μL of FeCl_3_ (30 mM in 1.2 M HCl). The mixture was incubated at room temperature in darkness for 15 min, and finally 600 μL of the mixture was added to a tube with 150 μL of trichloroacetic acid (10% wt/vol) to precipitate protein. The precipitated protein was removed by centrifugation at 10,000 g for 5 min, and absorbance at 670 nm of the resulting supernatant (200 μL) was determined. The H_2_S concentration of each sample was calculated against a calibration curve of NaHS.

### Small molecule studies

CBSB-S1 injected or *cbsb* CRISPR embryos were treated with either 1 mM betaine or 1 mM GYY4137 or 1 mM NaHS at 6 hpf. All embryos were then developed to 30 or 52 hpf in the 28°C incubator. Pictures were taken as described earlier.

### CRISPR studies

CRISPR/CAS9 target sequences were designed using E-CRISP. (Heigwer et al., 2014). pT3TS-nCas9n was a gift from Wenbiao Chen (Addgene plasmid # 46757) (Jao et al., 2013). sgRNA and capped *cas9* mRNA were synthesized by *in-vitro* transcription as previously described. (Varshney et al., 2015) The *cbsb* sgRNA was validated by the Guide-it™ sgRNA Screening Kit from Clontech. One-cell stage embryos were injected with 250 pg of sgRNA and 250 pg of *cas9* mRNA. Injected founder (F_0_) fish were fin-clipped at adulthood and screened for INDELs at the target location by Sanger sequencing. Two F_0_ founders containing the same 2 base pair deletion at the target site were bred to produce F_1_ mutants (lines C, H, and F). F_1_ mutants were raised to adulthood and in-crossed for production of F_2_ embryos, for which phenotypes were observed and quantified. For rescue, CRISPR embryos were placed in 1 mM betaine at 6 hpf. CRISPR phenotypes were evaluated at 5 dpf. All CRISPR lines were generated in the background of (Tg: *flk*:EGFP) (Choi et al., 2007) and CASPER (Zebrafish Information Resource Center), and is referred to as *flk*:EGFP CASPER in this study.

### Quantification details and statistics

#### Embryo length determination

Body length was measured from the tip of the head to the end of the trunk. Scale bar calibration and length measurements were done with a Leica MZFL III microscope using a stage micrometer and Q-Capture PRO 7 software. *T*-test was performed using graph pad prism program on **Figures 2E**, **3F**, **4D**, **5**, **6**. Data are plotted as mean values and error bars indicate standard error of the mean. The standard *t*-test was used to evaluate statistical significance. A *p*-value < 0.05 was considered significant and *p*-values < 0.01, 0.001, 0.0001 were considered very significant.

## Results

### Expression analysis of *cbsa* and *cbsb* genes during embryonic development

**Figure 1:**
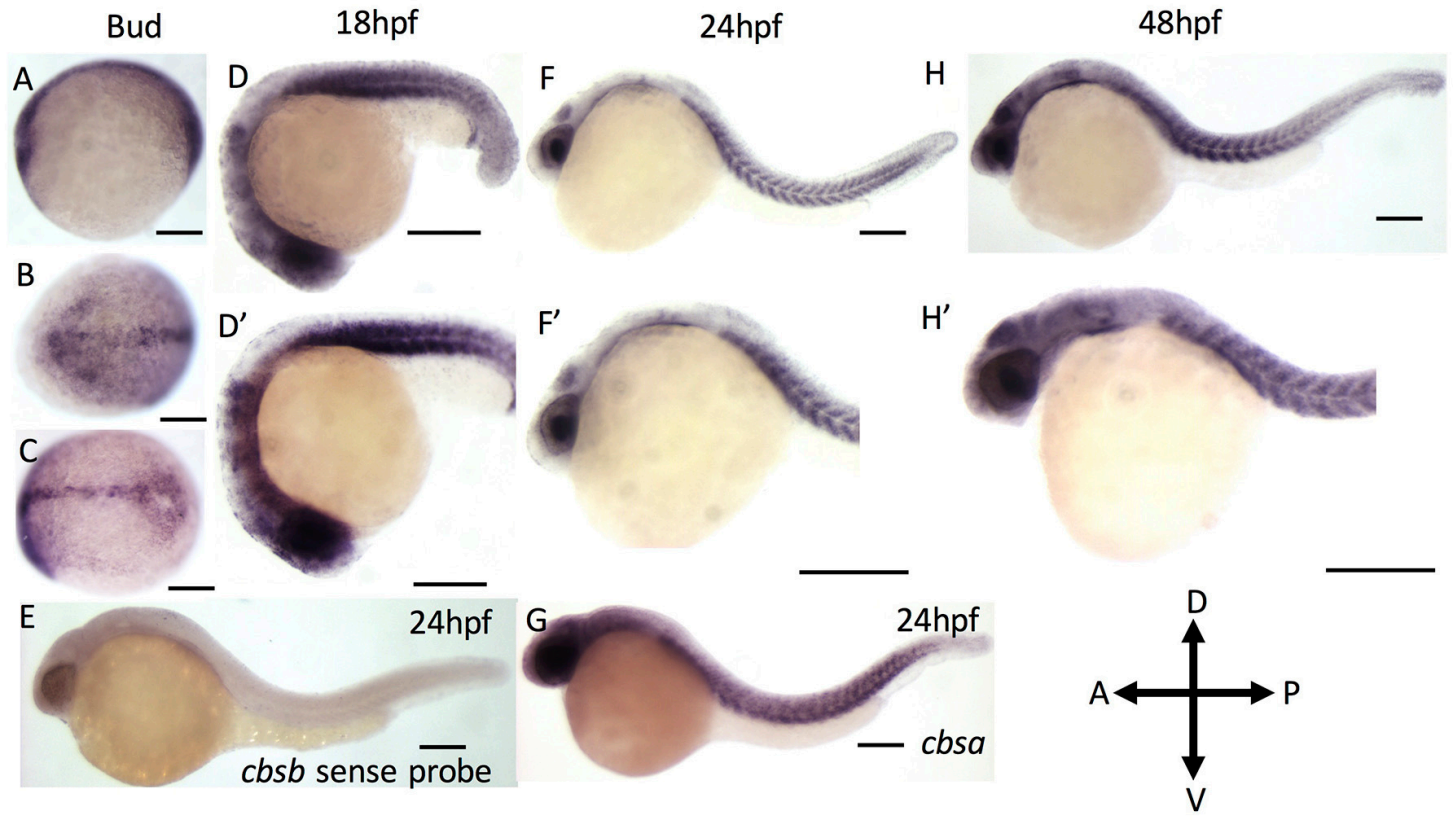
ISH analysis of *cbsa* and *cbsb* genes in zebrafish embryos. **(A–G)** are digioxigenin-labeled whole mount *in situ* hybridization for *cbsa* and *cbsb* RNA. Panels **(A–C)** are bud stage, (**D,F,H)** are 18, 24, and 48 hpf embryos respectively, Panels **(D–H**′**)** are high power images of the respective **(D,F,H)**. Panel **(E)** is a *cbsb* sense probed 24 hpf embryo, and Panel **(G)** is a *cbsa* antisense probed embryo at 24 hpf. Scale bars indicate 0.25 mm.

We first performed reverse transcription-based PCR (RT-PCR) using gene-specific primers for *cbsa, cbsb* and *actin* genes using RNA isolated from 2, 6, 10, 18, 24, 28, 36, 48, and 72 hpf embryos (Figure S1). Both *cbsa* and *cbsb* genes are maternally expressed and showed expression in 2 hpf embryo, which continued throughout all stages of development. The *cbsa* transcript is initially expressed minimally, but its expression begins to increase around 18 hpf (Figure S1). Notably, the *cbsb* expression peaks at end of gastrulation (10 hpf) stage, and the expression is constitutive in the remaining period of development (Figure S1). To detect the location of *cbsb* transcripts, we performed WISH using digoxigenin-labeled sense and antisense RNA probes at the bud stage, 18, 24, and 48 hpf (Figure 1). *Cbsb* expression is noticeably visible in the embryonic axis in the early tail bud stage (Figures 1A–C), an early indication of the importance of *cbsb* in axis development. At 18 hpf, *cbsb* expression is visible in the head region in the entire brain (Figure 1D) and in the somites in the body axis (Figure 1D′). At 24 hpf (Figures 1F,F′), *cbsb* expression pattern continues in the somites (Figure 1F), while in the head, expression shifts from the brain to the 4th ventricle highlighting the dynamic role of *cbsb* in early brain development. At this stage, sense probe controls do not show staining (Figure 1E). At 48 hpf, the expression of *cbsb* continues in both the brain and the 4th ventricle in the head as well as the somites in the trunk (Figures 1H,H′). WISH for *cbsa* was also performed at 24 hpf, a stage at which expression for *cbsa* was robust in RT-PCR results (Figure S1). *Cbsa* gene shows strong expression in the entire head, and in the trunk region (Figure 1G), particularly in the somites. Taken together, the expression data suggests that *cbsa* and *cbsb* genes are ubiquitously expressed throughout the head and in the somites, and suggest a role in head and axis development.

### Loss of function of *cbsb* but not *cbsa* affects embryonic axis development

**Figure 2:**
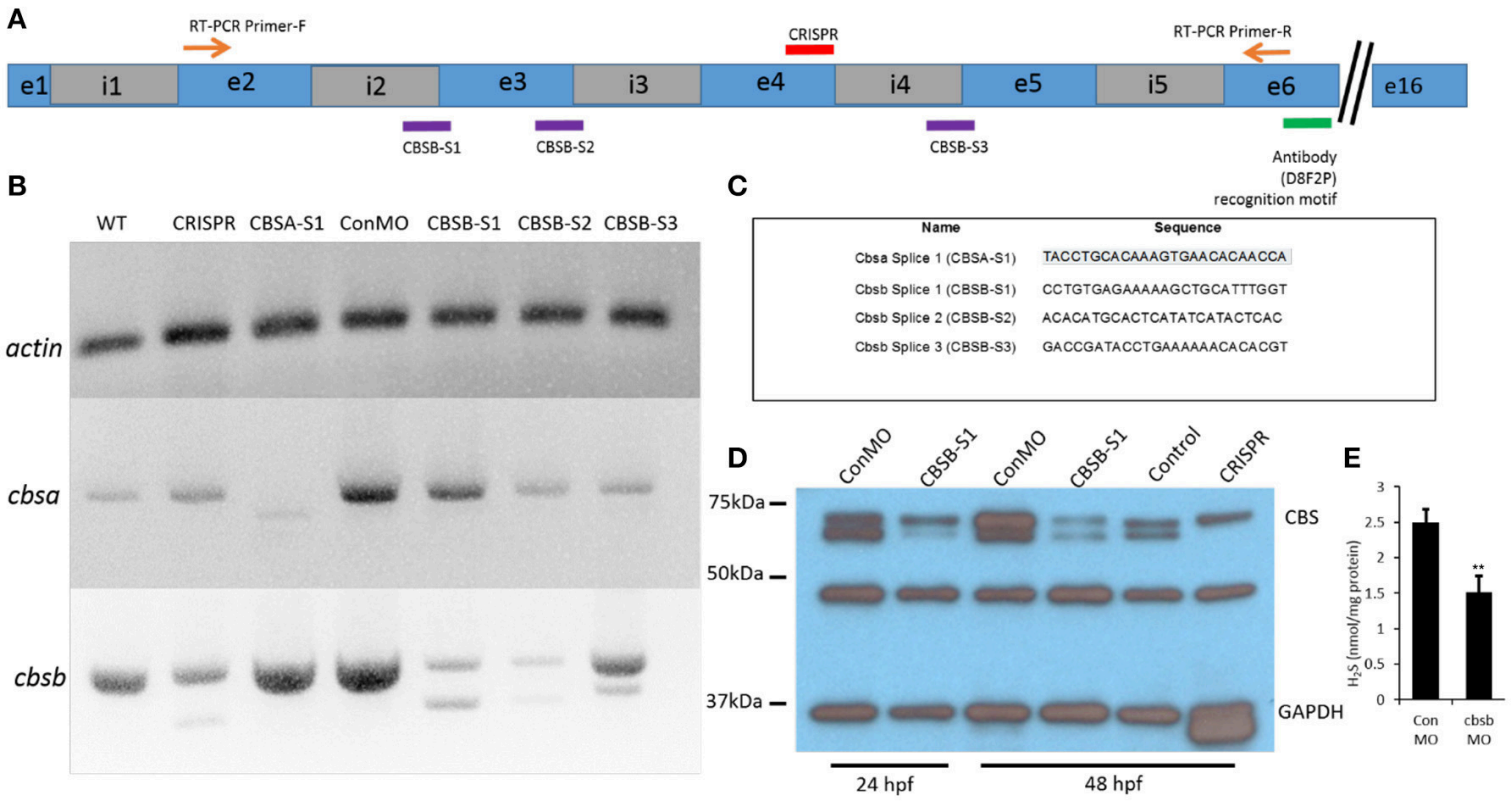
Loss-of-function efficacy studies. Panel **(A)** shows a “partial” cartoon representation of the *cbsb* genomic site with the location of the MO sites (purple rectangles) at appropriate intron (i) and exon (e) junctions, CRISPR-targeted site (red rectangle), site of RT-PCR forward **(F)** and reverse primers, and the antibody recognition site. Panel **(B)** shows RT-PCR for three genes (*cbsb, cbsa, actin*) in total RNA from injected embryos (~24 hpf) (left to right): wild type (WT) control and *cbsb* CRISPR-injected fish, *cbsa* splice 1 (CBSA-S1), control morpholino (ConMO), *cbsb* splice1 (CBSB-S1), *cbsb* splice 2 (CBSB-S2), *cbsb* splice 3 (CBSB-S3). Panel **(C)** shows the sequence of the morpholinos used in this study. Panel **(D)** shows CBS and GAPDH western blots for ConMO, CBSB-S1 at 24 and 48 hpf along with control and *cbsb* CRISPR fish at 48 hpf. Panel **(E)** shows the comparison between CBSB-S1 MO and ConMO-injected embryos for hydrogen sulfide production. *n* = 3 for both groups (data from three experiments). Twenty embryos in each group in each experiment. ^**^P < 0.01.

To investigate function of *cbsa* and *cbsb*, we employed two strategies, morpholinos (MOs) and CRISPR/Cas9 genomic engineering methods. We designed MOs that targeted *cbsa* and *cbsb* (Figure 2A) genes at specific splice junctions [*cbsa*: intron 4-exon 5 (CBSA-S1); *cbsb*: intron 2-exon3 (CBSB-S1), exon3-intron3 (CBSB-S2), and intron4-exon5 (CBSB-S3)]. MO sequences are provided (Figure 2C). Efficacy of MOs were determined at the RNA (Figure 2B) and protein (Figure 2D) level. CBSA-S1 indeed induced an alteration in *cbsa* transcript with no change in *cbsb* transcript (Figure 2B). Of the three splice MOs for *cbsb*, CBSB-S1 injected embryos produced a smaller alternate band (Figure 2B). The band was extracted and sequenced, which revealed that exon 3 was missing (data not shown). Importantly, *cbsa* mRNA in CBSB-S1-injected embryos did not show consistent change relative to WT or conMO lanes (Figure 2B, and data not shown). Similarly, CBSB-S2 and -S3 MOs did target *cbsb* and not *cbsa* (Figure 2B), but concentrations of MOs required to achieve this effect was high. Therefore, for *cbsb*, we eventually settled on CBSB-S1 MO, which produced consistent loss of native *cbsb* full-length transcript. We also investigated protein changes in the embryo lysates, and observed consistent down regulation of CBSB protein at 24 and 48 hpf (Figure 2D). Because CBS is an enzyme the catalyzes the production of H_2_S gas via a reaction where cysteine combines with homocysteine to form cystathionine (Wallace and Wang, 2015), we measured H_2_S levels in CBSB-S1 MO-injected and control MO (ConMO)-injected embryos. CBSB-S1 MO1 knockdown embryos showed reduction in H_2_S levels compared to their control counterparts (Figure 2E, ^*^p < 0.01).

**Figure 3:**
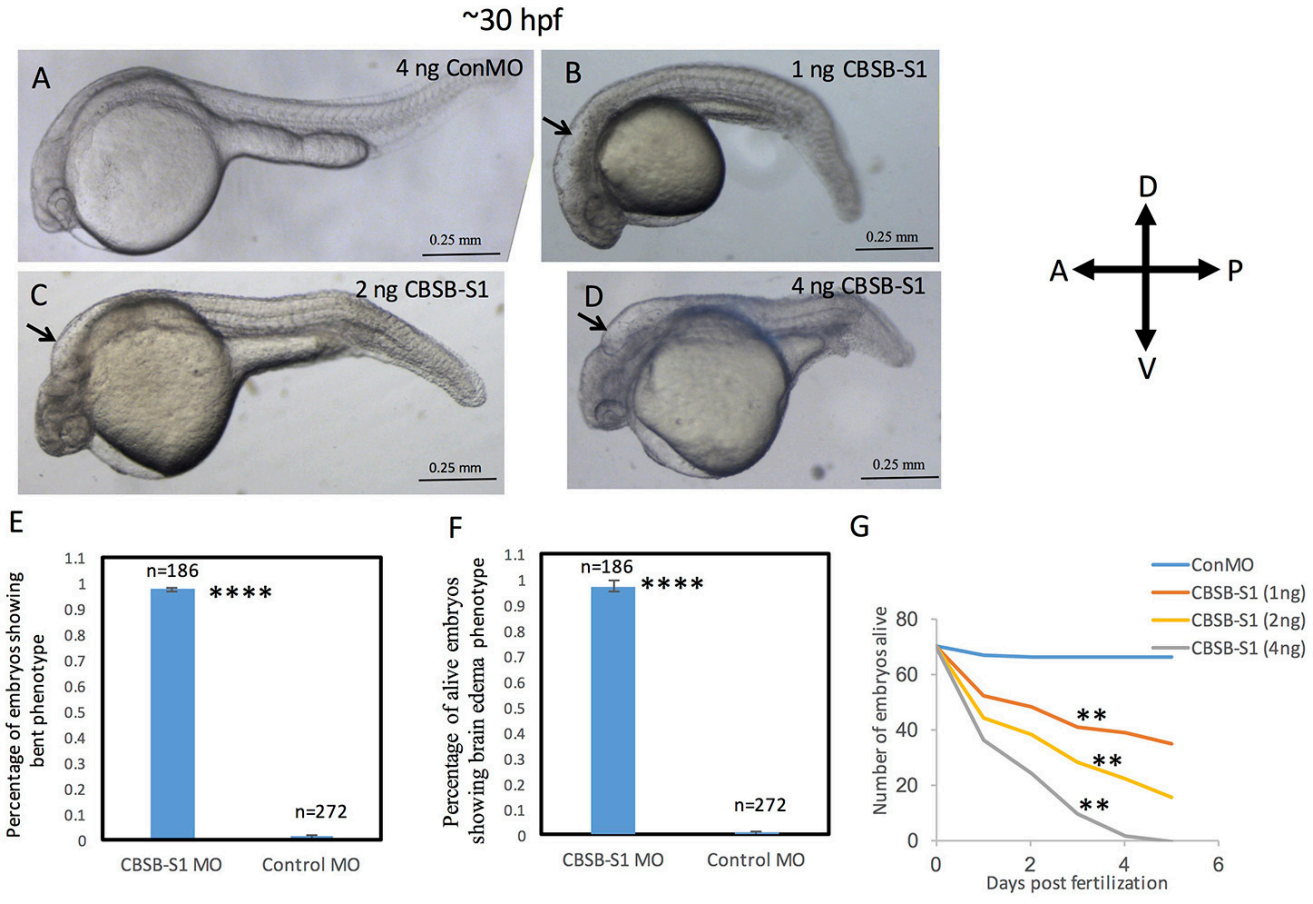
Dose-dependent effect of *cbsb* morpholinos at 30 hpf. Phase contrast images of 30 hpf embryos injected with 4 ng of control (Con) MO **(A)**; 1 ng *cbsb* splice1 (CBSB-S1) MO **(B)**; 2 ng (CBSB-S1) **(C)** and 4 ng (CBSB-S1) **(D)** are shown. Arrow shows brain edema. Panels **(E,F)** quantifies the number of CBSA-S1 MO-injected embryos displaying bent axis, and brain edema (black arrows) compared to ConMO-injected embryos. *n* = 186 surviving embryos for CBSB-S1 MO and *n* = 272 surviving embryos for ConMO, which were a total of four experiments ^****^P < 0.0001 for both bent axis phenotype and brain edema phenotype. **(G)** Percentage survival observed over a 5 day period for three different doses of CBSB-S1-injected (1 ng, *n* = 70; 2 ng, *n* = 70; 4 ng, *n* = 70) and ConMO-injected embryos (4 ng, *n* = 70), *n* = 6 time points, ^**^P < 0.01 for comparison between ConMO and each of the respective CBSB-S1 MO doses. Scale bars indicate 0.25 mm.

**Figure 4:**
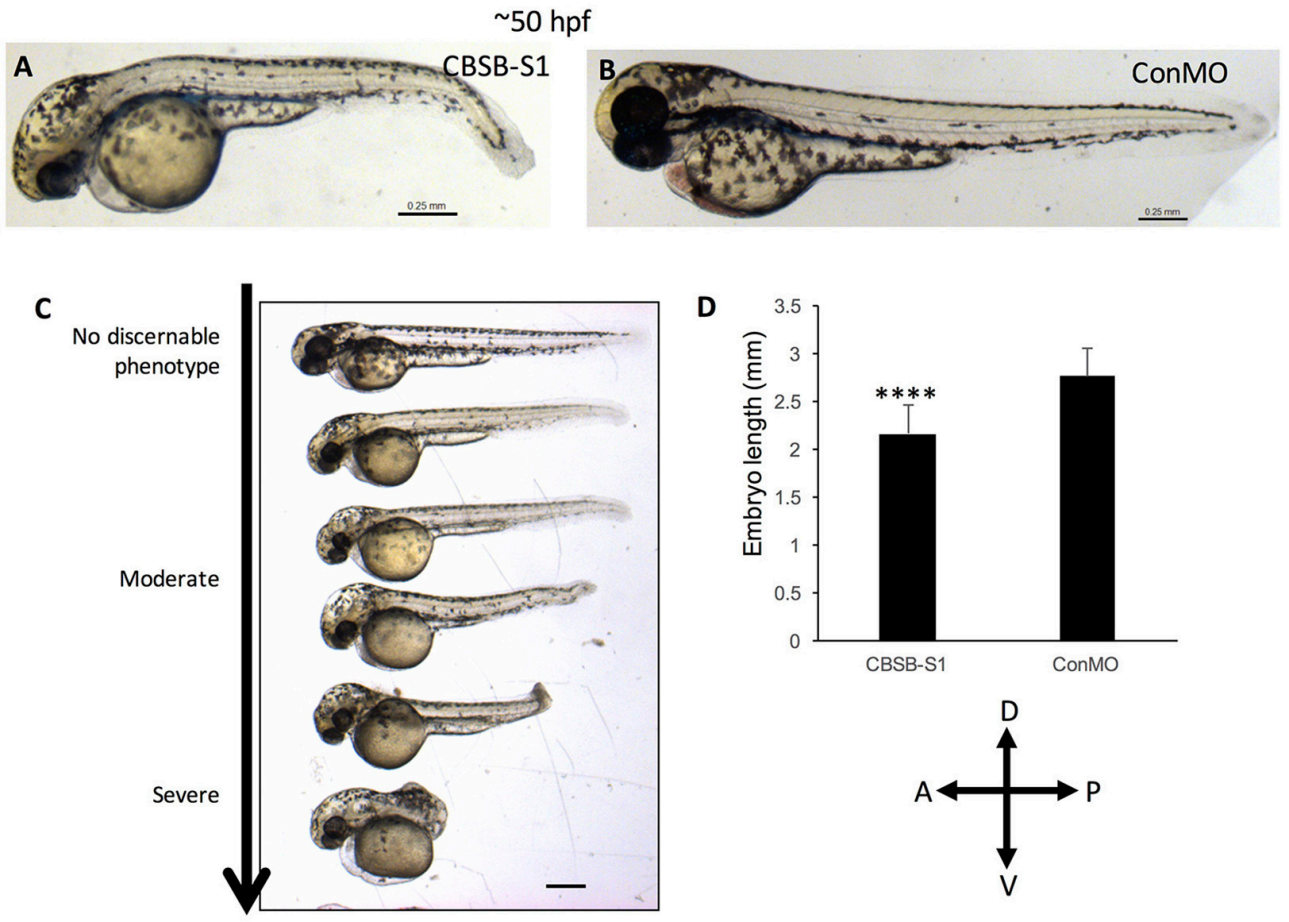
*cbsb* morphant phenotype at 52 hpf. Low magnification images of 52 hpf Tg (*flk*:EGFP) embryos injected with 2 ng CBSB-S1 **(A)** and 2 ng control (Con) MO are shown. Panel **(C)** shows various phenotypes observed from mild to server for CBSB-S1 at 52 hpf. Note the progressive loss of axis length. Panel **(D)** shows quantification of the length of the axis is shown in **(A)**. Y-axis shows mean length of the embryo measured as the longest horizontal dimension between the start of the yolk and the end of the tail (*n* = 11 for CBSB-S1 and ConMO-injected embryos), ^****^P < 0.0001. In the figure panels, the embryo orientation is left is anterior (A) and right is posterior (P) while top is dorsal (D) and bottom is ventral (V). Scale bars indicate 0.25 mm.

The WISH analysis suggested redundant functions for *cbsa* and *cbsb* in embryonic development. CBSA-S1 alone injected embryos showed no phenotype (data not shown). CBSB-S1 when injected alone into 1-cell stage zebrafish embryos showed a shortened anterior-posterior axis and head edema (Figures 3B–D) compared to ConMO-injected embryos at 30 hpf (Figure 3A). Therefore, we concluded that all effects were observed due to *cbsb* loss-of-function. A dose-dependent increase in severity of the bent axis and brain edema phenotypes was observed (Figures 3B–D), which was quantified (bent axis, Figure 3E, ^****^P < 0.0001; brain edema, Figure 3F, ^****^P < 0.0001), and correlated well with percentage mortality (Figure 3G, ^**^P < 0.01). At ~50 hpf, the axis defects were pronounced in CBSB-S1-injected embryos (Figure 4A) compared to ConMO-injected embryos (Figure 4B), and phenotype expressivity varied from moderate to severe phenotypes (Figure 4C and Figure S2). The axis length was measured and showed decrease in CBSB-S1-injected embryos compared to controls (Figure 4D, ^****^P < 0.0001). We also investigated the vasculature grossly since we injected CBSB-S1 into transgenic embryos where VEGFR2 promoter or fms-like kinase (FLK) drives enhanced green fluorescent protein (EGFP) [Tg (*flk:*EGFP)] expression (Choi et al., 2007) in the vasculature. We did not observe much changes to the major vessels in CBSB-S1-injected embryos compared to ConMO-injected embryos (Figure S3). These results suggest that *cbsb* is important for axis development.

### *cbsb* CRISPR-modified embryos phenocopy *cbSb* MO

**Figure 5:**
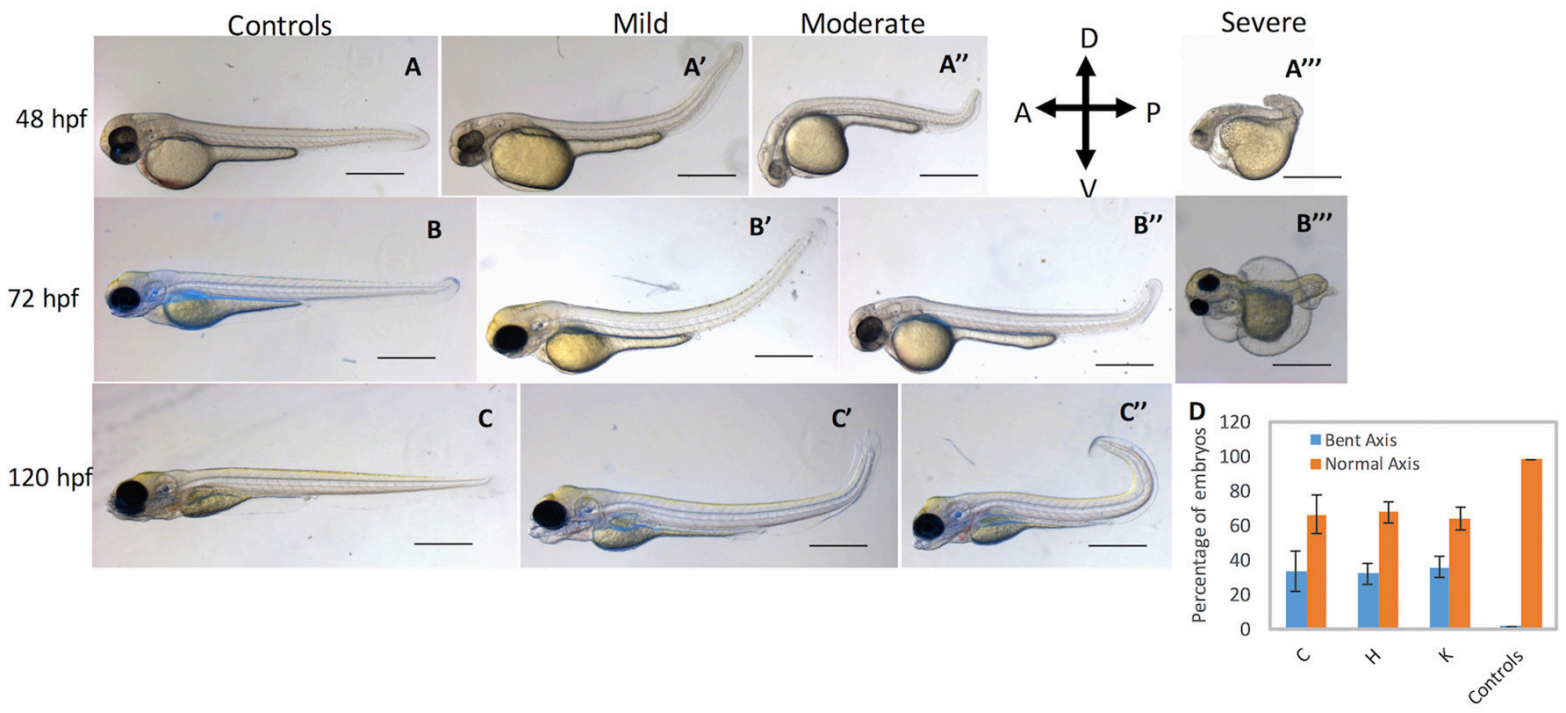
*cbsb* CRISPR embryos show axis defects. Control *flk*:EGFP CASPER embryos are shown at 48 hpf **(A)**, 72 **(B)**, and 120 hpf **(C)**. *cbsb* CRISPR embryos are shown at 48 hpf **(A**′**-A**‴**)**, 72 hpf **(B**′**-B**‴**)**, and 120 hpf **(C**′**,C**″**)**. Mild, medium, and severe phenotypes are shown for each time point for the *cbsb* CRISPR embryos except at 120 hpf. Panel **(D)** shows quantification of the axis defects in three independent CRISPR lines (C, H, and K) and comparison of the axis defects with control *flk*:EGFP CASPER line. Y axis shows the average of the percentage of embryos from five experiments with bent axis and normal axis. *n* = 311 embryos for C line, *n* = 236 embryos for H line, *n* = 241 embryos for K line and *n* = 311 embryos for the control *flk*:EGFP CASPER line which are total number of embryos from five independent experiments. To perform statistical analysis, embryos showing any degree of bent axis phenotype (mild, medium, or severe) have been grouped together into one category called bent axis. ^*^P < 0.05 for comparison of percent embryos with bent axis between controls and CRISPR line C, ^***^P < 0.001 for comparison of percent embryos with bent axis between controls and CRISPR line H and ^***^P < 0.001 for comparison of percent embryos with bent axis between controls and CRISPR line K. In the figure panels, the embryo orientation is left is anterior (A) and right is posterior (P) while top is dorsal (D) and bottom is ventral (V). Scale bars in the 48 and 72 hpf panels indicate 0.25 mm; scale bars in 120 hpf panels **(C–C**″**)** are 0.4 mm. Error bars indicate standard error of the mean of five experiments.

To complement MO loss-of-function analysis, we generated guide RNAs (gRNAs) that target exon 4 of *cbsb* gene (Figure 4A and Figure S4A). Guide RNA efficacy was demonstrated (Figure S4B), and gRNA + Cas9 injected fish were genotyped for genomic modifications (Figure S4C). Indeed, 35.4% of the gRNA/Cas9-injected F0 fish showed 2 bp deletions, and several showed deletions and point mutations (Figure S4C). Also, *cbsb* RNA was lower and no change in *cbsa* RNA was observed in *cbsb* CRISPR fish when compared to control fish (Figure 2B). The CBS protein level was lower (Figure 2D) in *cbsb* CRISPR fish compared to control fish. We raised the F0 *cbsb* CRISPR fish and generated three independent *cbsb* CRISPR fish lines (C, H, and K). Three distinct axis phenotypes were observed (Figure 5), which were categorized as mild, medium and severe. Phenotypes were readily visible at 48 hpf with a bent axis and tail. In extreme cases, the axis was severely truncated (Figure 5, severe phenotype) similar to CBSB-S1 embryos (Figure S2). The phenotype progressed and became evident by 72 and 120 hpf. It is noteworthy that the bent in the axis resulted in alterations of tail to upward or downward orientation. Quantification of the axis phenotype severity for CRISPR fish in three independent lines (Figure 5D) showed ~30% of defective embryos per line. Based on *t*-test analysis of the data from 5 independent experiments (Figure 5D), the differences between the percentage of embryos with bent axis in each of the *cbsb* CRISPR lines (C, ^*^P < 0.05; H and K, ^***^P < 0.001) was found to be statistically significant compared to the percentage of embryos with bent axis in the control *flk*:EGFP CASPER line. These results show that *cbsb* is mainly responsible for axis development in zebrafish.

### Rescue of *cbsb* axis phenotype with small molecules

**Figure 6:**
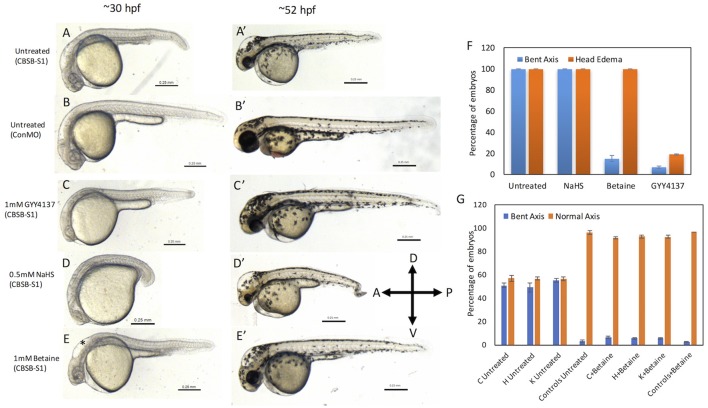
Rescue of *cbsb* phenotypes using small molecules at 30 and 52 hpf. All embryos were injected to 2 ng of cbsb-splice1 (CBSB-S1) or control (Con) morpholinos (MOs). Panels **(A–E)** are 30 hpf phase contrast images, and corresponding **(A**′**-E**′**)** are 52 hpf images. Panels **(A,A**′**)** is an untreated embryo injected with CBSB-S1 MO, which shows distinct phenotypes including head edema, and axis defect. Panels **(B,B**′**)** are ConMO-injected embryo. Panels **(C,C**′**)** are CBSB-S1-injected embryo treated with 1 mM GYY4137, which rescues the head edema and axis defects. Panels **(D,D**′**)** are CBSB-S1-injected embryo treated with 0.5 mM NaHS, which does not rescue the phenotype. Panels **(E,E**′**)** are CBSB-S1 injected embryo treated with 1 mM betaine, which rescues the axis defect but not the head edema (black asterisk). Panel **(F)** shows quantification of the rescue of bent axis and head edema phenotypes for CBSB-S1 MO-injected embryos. Y axis shows percentage of embryos with bent axis phenotypes and head edema phenotypes (average of three experiments). Three experiments were performed. X axis categories: Untreated *n* = 118 embryos, NaHS treated *n* = 111 embryos, Betaine treated *n* = 120 embryos and GYY4137 treated *n* = 121 embryos which are total number of embryos for 3 experiments. ^***^P < 0.0001 for comparison between untreated and betaine treated for bent axis phenotypes. ^***^P < 0.0001 for comparison between untreated and GYY4137 treated for both bent axis and head edema phenotypes. Error bars indicate standard error of the mean of three experiments. Panel **(G)** shows quantification of *cbsb* CRISPR lines (C, H, and K) for the betaine rescue of axis defects at 5 dpf. For statistical analysis, all embryos with bent axis (irrespective of severity: mild, medium, and severe) were placed into one category bent axis. Y axis shows average of percentage of embryos (with bent axis or normal axis) from three experiments. *n* = 114 For C Untreated, *n* = 120 for H Untreated, *n* = 112 for K Untreated, *n* = 121 for Controls Untreated, *n* = 120 for C + Betaine, *n* = 119 for H + Betaine, *n* = 124 for K + Betaine and *n* = 117 for Controls + Betaine, which are total number of embryos from 3 independent experiments. *T*-test analysis resulted in ^**^P < 0.001 for comparisons between untreated *cbsb* CRISPR lines and *cbsb* CRISPR lines treated with betaine. (C vs. C + Betaine, H vs. H + Betaine, K vs. K + Betaine). Differences between control and control + Betaine groups were not statistically significant. Error bars indicate standard error of the mean of three experiments.

Because CBSB is a critical enzyme in cysteine & H_2_S metabolism (Figure S5), we investigated whether small molecules that influence these pathways would rescue the axis phenotype. We treated CBSB-S1-injected embryos with small molecules that rescue loss or gain of specific products in the CBSB enzyme pathway. Hydrogen sulfide (H_2_S) is an important byproduct of cysteine metabolism, and was observed to be lower in CBSB-S1 knockdown embryos (Figure 2E). We treated CBSB-S1-injected embryos at 6 hpf with two H_2_S donor compounds [Sodium hydrosulfide (NaHS), & GYY4137 (Figure S6)], and investigated whether replenishment of H_2_S would rescue the *cbsb* morphant phenotype. NaHS is an immediate H_2_S releasing compound while GYY4137 is a slow-releasing H_2_S donor. Intriguingly, GYY4137 (Figures 6C,C′) rescues the head edema and axis defects at 52 hpf while NaHS (Figure 6D) does not rescue these phenotypes suggesting that a long-term release of H_2_S is necessary for CBS enzyme function. *Cbs* deficiency also causes increase in homocysteine levels (Jhee and Kruger, 2005), Therefore, drug betaine that lowers homocysteine levels was tested, which rescued the axis defect at 52 hpf (Figure 6E′) but not the head edema (Figure 6E, black asterisk). This result along with the GYY4137 result suggests that embryonic axis defect is sensitive to loss of H_2_S and increase in homocysteine levels. Quantification is shown in Figure 6F.

Based on *t*-test analysis of data from 3 independent experiments (Figure 6F), the differences between untreated morphant embryos and morphant embryos treated with betaine were found to be statistically very significant (^***^P < 0.0001) for the bent axis phenotype. Similarly, the differences between untreated morphant embryos and morphant embryos treated with GYY4137 were found to be statistically very significant (^***^P < 0.0001) for both the bent axis phenotype and head edema phenotype. We also treated *cbsb* CRISPR fish lines with betaine (Figure 6G), which resulted in loss of severe and medium axis phenotype in all three lines, and reduction in the total % of embryos displaying phenotype. For Figure 6G, based on data from 3 independent experiments, *t*-test analysis showed that the differences between the untreated and treated groups for the three CRISPR lines (C Untreated compared to C+Betaine; H Untreated compared to H+Betaine; K Untreated compared to K+Betaine) were statistically very significant (^***^P < 0.001). The differences between the Controls Untreated and Controls + Betaine groups were not statistically significant.

## Discussion

This manuscript reports the importance of Cbsb enzyme in maintaining H_2_S homeostasis during embryonic zebrafish development. The salient features include, the characterization of the expression of *cbsa* and *cbsb* transcripts during embryonic development, the loss-of-function analysis using two complementary independent approaches, and finally the rescue of the axis phenotype with small molecules that are used for treatment of CBSB enzyme deficiency in the clinic.

The RNA expression pattern for *cbsa* and *cbsb* across developmental stages shows that *cbsb* is the dominant gene in terms of its levels, which later also is the case for function. The expression of *cbsb* is observed as early as bud stage (10 hpf) along the developing embryonic axis, and continues through 24 and 48 hpf along the anterior-posterior axis of the embryo. *Cbsb* expression is prominent in the brain and somite muscle, two tissues that are highly susceptible to changes in H_2_S levels during normal physiology.

Our approach to assess function of *cbsb* was to use complementary methods such as MOs and CRISPR-Cas9. Given the evidence that MOs induced phenotypes do not replicate fully in genetic mutants (Kok et al., 2015), we only focused on phenotypes that were observed routinely under both loss-of-function conditions. One main difference between the two approaches is that the splice1 MO (CBSB-S1) targets exon 3 while *cbsb* CRISPR targets exon 4 of *cbsb* gene. In the morphants, we did observe head edema and axis defect. Because *cbsb* CRISPR embryos only showed anterior-posterior shorter axis defect, we reasoned that axis defect as the primary defect for loss of *cbsb* function during development. It is reasonable to argue that head edema is a secondary effect related to extremely high levels of homocysteine (Figure S7) and methionine in the brain, which is presumed to occur with CBS enzyme deficiency (Figure S5). However, our results and those from others collectively argue against the secondary effect possibility. For example, patients with *CBS* deficiency who were treated with betaine therapy showed cerebral edema (Yaghmai et al., 2002). Consistent with this observation, treatment of CBSB-S1 embryos with betaine, a molecule known to reduce homocysteine levels and increase methionine levels in plasma failed to rescue head edema (Figure 6F). Thus, the edema seems to be an upstream effect of homocysteine levels in the brain, perhaps increased methionine. Homocysteine and methionine are also products of folate-mediated one carbon cycle, and evidence exists in zebrafish that folate pathway disruption in zebrafish (Lee et al., 2012) causes developmental delays including shortened axis development. Folate also known as vitamin B_9_ is a co-factor for enzymes involved in transmethylation reactions and *de novo* synthesis of purines and thymidylate. These products are critical for DNA replication and thus cell cycle-associated events in a developing embryo. Pathway disruption in folate leads to disruption in thymidylate levels, which in turn affects dTTP thus lengthening the cell cycle in the developing embryo (Lee et al., 2012). This delay in S-phase along with the observed increase in apoptosis in these embryonic cells in zebrafish results in fewer cells in the embryo that are necessary for organism development including axis formation. Our results suggest that alteration of products (homocysteine and methionine) shared by the transsulfuration and folate pathways predictably show similar consequence in a developing embryo, namely shortened axis development. This interconnection provides one mechanistic explanation to our findings, and requires further exploration in the context of embryonic development.

Previous work has shown that small molecule slow releasing H_2_S donor GYY4137 treated for 4 days in zebrafish larvae significantly reduced sodium uptake (Kumai et al., 2015), and this reduced sodium uptake was rescued when CBS or CSE was pharmacologically inhibited. In our study, GYY4137 rescues both axis and edema defects associated with loss of *cbsb*. Taken together, this implies that GYY4137 influences CBSB enzyme directly or indirectly to influence sodium uptake in cells of a developing embryo, and this influence is critical for restoring homeostasis in embryo, which includes proliferation and apoptosis of cells that are necessary for axis formation. In addition to H_2_S, gases such as nitric oxide (NO), and carbon monoxide (CO) collectively referred to as gasotransmitters interact leading to oxidative stress in the developing embryo (Olson et al., 2012). These gases have already been implicated in control of breathing and ion regulation in fish (Perry et al., 2016). NO also has profound effects on neuromuscular properties in zebrafish (Jay et al., 2014), which directly correlates to trunk axis structural integrity. Thus, future work will focus on identifying the interactions of gasotransmitters and the mechanisms that they associate with in facilitating embryonic growth and development.

In summary, we have identified a novel phenotype for *cbsb* in zebrafish using multiple approaches, and have demonstrated that *cbsb* is partly responsible for anterior-posterior axis development in zebrafish. A combination of homocysteine and H_2_S regulation is responsible for the axis development, and structures along the axis of the embryos are highly susceptible to this regulation.

## Author contributions

SP designed and performed experiments, interpreted data, wrote, and edited the manuscript; CK designed and performed experiments, and interpreted data, wrote, and edited the manuscript; AD designed and performed experiments, and interpreted data; SE-B designed and performed experiments, and interpreted data; NL performed experiments and interpreted data; RB provided intellectual input, direction in experimental design, and edited the manuscript; PM provided intellectual input, direction in experimental design, provided resources, and edited the manuscript; RR provided intellectual input, direction in experimental design, provided resources, and wrote the manuscript.

### Conflict of interest statement

The authors declare that the research was conducted in the absence of any commercial or financial relationships that could be construed as a potential conflict of interest.

## References

[B1] Bedell V. M. Yeo S. Y. Park K. W. Chung J. Seth P. Shivalingappa V.. (2005). Roundabout4 is essential for angiogenesis *in vivo*. Proc. Natl. Acad. Sci. U.S.A. 102, 6373–6378. 10.1073/pnas.0408318102 15849270 PMC1088354

[B2] Choi J. Dong L. Ahn J. Dao D. Hammerschmidt M. Chen J. N. (2007). FoxH1 negatively modulates flk1 gene expression and vascular formation in zebrafish. Dev. Biol. 304, 735–744. 10.1016/j.ydbio.2007.01.023 17306248 PMC1876740

[B3] Dayal S. Bottiglieri T. Arning E. Maeda N. Malinow M. R. Sigmund C. D.. (2001). Endothelial dysfunction and elevation of S-adenosylhomocysteine in cystathionine beta-synthase-deficient mice. Circ. Res. 88, 1203–1209. 10.1161/hh1101.092180 11397788

[B4] Ekker S. C. (2000). Morphants: a new systematic vertebrate functional genomics approach. Yeast 17, 302–306. 10.1002/1097-0061(200012)17:4<302::AID-YEA53>3.0.CO;2-# 11119307 PMC2448384

[B5] Födinger M. Buchmayer H. Sunder-Plassmann G. (1999). Molecular genetics of homocysteine metabolism. Miner. Electrolyte Metab. 25, 269–278. 10.1159/000057459 10681651

[B6] Ganapathy P. S. Moister B. Roon P. Mysona B. A. Duplantier J. Dun Y.. (2009). Endogenous elevation of homocysteine induces retinal neuron death in the cystathionine-beta-synthase mutant mouse. Invest. Ophthalmol. Vis. Sci. 50, 4460–4470. 10.1167/iovs.09-3402 19357353 PMC2756015

[B7] Hamelet J. Demuth K. Paul J. L. Delabar J. M. Janel N. (2007a). Hyperhomocysteinemia due to cystathionine beta synthase deficiency induces dysregulation of genes involved in hepatic lipid homeostasis in mice. J. Hepatol. 46, 151–159. 10.1016/j.jhep.2006.07.028 17030070

[B8] Hamelet J. Maurin N. Fulchiron R. Delabar J. M. Janel N. (2007b). Mice lacking cystathionine beta synthase have lung fibrosis and air space enlargement. Exp. Mol. Pathol. 83, 249–253. 10.1016/j.yexmp.2007.04.005 17543941

[B9] Heigwer F. Kerr G. Boutros M. (2014). E-CRISP: fast CRISPR target site identification. Nat. Methods 11, 122–123. 10.1038/nmeth.2812 24481216

[B10] Hruscha A. Krawitz P. Rechenberg A. Heinrich V. Hecht J. Haass C.. (2013). Efficient CRISPR/Cas9 genome editing with low off-target effects in zebrafish. Development 140, 4982–4987. 10.1242/dev.099085 24257628

[B11] Huang C. W. Moore P. K. (2015). H2S synthesizing enzymes: biochemistry and molecular aspects. Handb. Exp. Pharmacol. 230, 3–25. 10.1007/978-3-319-18144-8_1 26162827

[B12] Jao L. E. Wente S. R. Chen W. (2013). Efficient multiplex biallelic zebrafish genome editing using a CRISPR nuclease system. Proc. Natl. Acad. Sci. U.S.A. 110, 13904–13909. 10.1073/pnas.1308335110 23918387 PMC3752207

[B13] Jay M. Bradley S. Mcdearmid J. R. (2014). Effects of nitric oxide on neuromuscular properties of developing zebrafish embryos. PLoS ONE 9:e86930. 10.1371/journal.pone.0086930 24489806 PMC3904980

[B14] Jhee K. H. Kruger W. D. (2005). The role of cystathionine beta-synthase in homocysteine metabolism. Antioxid. Redox Signal. 7, 813–822. 10.1089/ars.2005.7.813 15890029

[B15] Kok F. O. Shin M. Ni C. W. Gupta A. Grosse A. S. Van Impel A.. (2015). Reverse genetic screening reveals poor correlation between morpholino-induced and mutant phenotypes in zebrafish. Dev. Cell 32, 97–108. 10.1016/j.devcel.2014.11.018 25533206 PMC4487878

[B16] Kraus J. P. Janosík M. Kozich V. Mandell R. Shih V. Sperandeo M. P.. (1999). Cystathionine beta-synthase mutations in homocystinuria. Hum. Mutat. 13, 362–375. 10.1002/(SICI)1098-1004(1999)13:5<362::AID-HUMU4>3.0.CO;2-K 10338090

[B17] Kumai Y. Porteus C. S. Kwong R. W. Perry S. F. (2015). Hydrogen sulfide inhibits Na+ uptake in larval zebrafish, *Danio rerio*. Pflugers Arch. 467, 651–664. 10.1007/s00424-014-1550-y 24939700

[B18] Kwong R. W. Perry S. F. (2015). Hydrogen sulfide promotes calcium uptake in larval zebrafish. Am. J. Physiol. Cell Physiol. 309, C60–69. 10.1152/ajpcell.00053.2015 25948733 PMC4490323

[B19] Lee M. S. Bonner J. R. Bernard D. J. Sanchez E. L. Sause E. T. Prentice R. R.. (2012). Disruption of the folate pathway in zebrafish causes developmental defects. BMC Dev. Biol. 12:12. 10.1186/1471-213X-12-12 22480165 PMC3410756

[B20] Lentz S. R. Erger R. A. Dayal S. Maeda N. Malinow M. R. Heistad D. D.. (2000). Folate dependence of hyperhomocysteinemia and vascular dysfunction in cystathionine beta-synthase-deficient mice. Am. J. Physiol. Heart Circ. Physiol. 279, H970–H975. 10.1152/ajpheart.2000.279.3.H970 10993757

[B21] MacLean K. N. Sikora J. KoŽich V. Jiang H. Greiner L. S. Kraus E.. (2010). A novel transgenic mouse model of CBS-deficient homocystinuria does not incur hepatic steatosis or fibrosis and exhibits a hypercoagulative phenotype that is ameliorated by betaine treatment. Mol. Genet. Metab. 101, 153–162. 10.1016/j.ymgme.2010.06.010 20638879 PMC2954364

[B22] Majtan T. Pey A. L. Ereño-Orbea J. Martínez-Cruz L. A. Kraus J. P. (2016). Targeting cystathionine beta-synthase misfolding in homocystinuria by small ligands: state of the art and future directions. Curr. Drug Targets 17, 1455–1470. 10.2174/1389450117666160302094910 26931358

[B23] Olson K. R. Donald J. A. Dombkowski R. A. Perry S. F. (2012). Evolutionary and comparative aspects of nitric oxide, carbon monoxide and hydrogen sulfide. Respir. Physiol. Neurobiol. 184, 117–129. 10.1016/j.resp.2012.04.004 22546339

[B24] Papapetropoulos A. Pyriochou A. Altaany Z. Yang G. Marazioti A. Zhou Z.. (2009). Hydrogen sulfide is an endogenous stimulator of angiogenesis. Proc. Natl. Acad. Sci. U.S.A. 106, 21972–21977. 10.1073/pnas.0908047106 19955410 PMC2799889

[B25] Perry S. Kumai Y. Porteus C. S. Tzaneva V. Kwong R. W. (2016). An emerging role for gasotransmitters in the control of breathing and ionic regulation in fish. J. Comp. Physiol. B Biochem. Syst. Environ. Physiol. 186, 145–159. 10.1007/s00360-015-0949-x 26660653

[B26] Porteus C. S. Abdallah S. J. Pollack J. Kumai Y. Kwong R. W. Yew H. M.. (2014). The role of hydrogen sulphide in the control of breathing in hypoxic zebrafish (*Danio rerio*). J. Physiol. 592, 3075–3088. 10.1113/jphysiol.2014.271098 24756639 PMC4214661

[B27] Postlethwait J. H. Woods I. G. Ngo-Hazelett P. Yan Y. L. Kelly P. D. Chu F.. (2000). Zebrafish comparative genomics and the origins of vertebrate chromosomes. Genome Res. 10, 1890–1902. 10.1101/gr.164800 11116085

[B28] Robert K. Maurin N. Ledru A. Delabar J. Janel N. (2004). Hyperkeratosis in cystathionine beta synthase-deficient mice: an animal model of hyperhomocysteinemia. Anat. Rec. A Discov. Mol. Cell. Evol. Biol. 280, 1072–1076. 10.1002/ar.a.20082 15386278

[B29] Robert K. Maurin N. Vayssettes C. Siauve N. Janel N. (2005). Cystathionine beta synthase deficiency affects mouse endochondral ossification. Anat. Rec. A Discov. Mol. Cell. Evol. Biol. 282, 1–7. 10.1002/ar.a.20145 15622513

[B30] Thisse B. Thisse C. (2014). *In situ* hybridization on whole-mount zebrafish embryos and young larvae. Methods Mol. Biol. 1211, 53–67. 10.1007/978-1-4939-1459-3_5 25218376

[B31] Varshney G. K. Pei W. Lafave M. C. Idol J. Xu L. Gallardo V.. (2015). High-throughput gene targeting and phenotyping in zebrafish using CRISPR/Cas9. Genome Res. 25, 1030–1042. 10.1101/gr.186379.114 26048245 PMC4484386

[B32] Vitvitsky V. Dayal S. Stabler S. Zhou Y. Wang H. Lentz S. R.. (2004). Perturbations in homocysteine-linked redox homeostasis in a murine model for hyperhomocysteinemia. Am. J. Physiol. Regul. Integr. Comp. Physiol. 287, R39–R46. 10.1152/ajpregu.00036.2004 15016621

[B33] Wallace J. L. Wang R. (2015). Hydrogen sulfide-based therapeutics: exploiting a unique but ubiquitous gasotransmitter. Nat. Rev. Drug Discov. 14, 329–345. 10.1038/nrd4433 25849904

[B34] Watanabe M. Osada J. Aratani Y. Kluckman K. Reddick R. Malinow M. R.. (1995). Mice deficient in cystathionine beta-synthase: animal models for mild and severe homocyst(e)inemia. Proc. Natl. Acad. Sci. U.S.A. 92, 1585–1589. 10.1073/pnas.92.5.1585 7878023 PMC42564

[B35] Yaghmai R. Kashani A. H. Geraghty M. T. Okoh J. Pomper M. Tangerman A.. (2002). Progressive cerebral edema associated with high methionine levels and betaine therapy in a patient with cystathionine beta-synthase (CBS) deficiency. Am. J. Med. Genet. 108, 57–63. 10.1002/ajmg.10186 11857551

