# Corrigendum: Cystathionine β-Synthase Is Necessary for Axis Development *in vivo*

**DOI:** 10.3389/fcell.2018.00121

**Published:** 2018-09-27

**Authors:** Shubhangi Prabhudesai, Chris Koceja, Anindya Dey, Shahram Eisa-Beygi, Noah R. Leigh, Resham Bhattacharya, Priyabrata Mukherjee, Ramani Ramchandran

**Affiliations:** ^1^Department of Pediatrics, Medical College of Wisconsin, Milwaukee, WI, United States; ^2^Department of Obstetrics and Gynecology, University of Oklahoma Health Science Center, Oklahoma City, OK, United States; ^3^Pediatrics Radiology, Developmental Vascular Biology Program, Children's Research Institute, Medical College of Wisconsin, Milwaukee, WI, United States; ^4^Milwaukee Health Department, City of Milwaukee, Milwaukee, WI, United States; ^5^Peggy and Charles Stephenson Cancer Center, University of Oklahoma Health Science Center, Oklahoma City, OK, United States; ^6^Department of Pathology, University of Oklahoma Health Science Center, Oklahoma City, OK, United States; ^7^Department of Cell Biology, University of Oklahoma Health Science Center, Oklahoma City, OK, United States; ^8^Obstetrics and Gynecology, Medical College of Wisconsin, Milwaukee, WI, United States

**Keywords:** zebrafish, CRISPR, small molecules, methionine, homcystinuria, hydrogen sulfide, morpholino

In the original article, there was an error in Figure 2C as published. The sequence of the *cbsa splice* 1 (CBSA-S1) morpholino was incorrectly typed as

TACCTGCACAAAGTGAACACACAACCA

The correct sequence is

TACCTGCACAAAGTGAACACAACCA

The name of the morpholino was changed in the figure from *cbsa splice* (CBSA-S1) to *cbsa splice* 1 (CBSA-S1) to match the legend.

The corrected Figure 2 appears below. The authors apologize for this error and state that this does not change the scientific conclusions of the article in any way.

**Figure 2:**
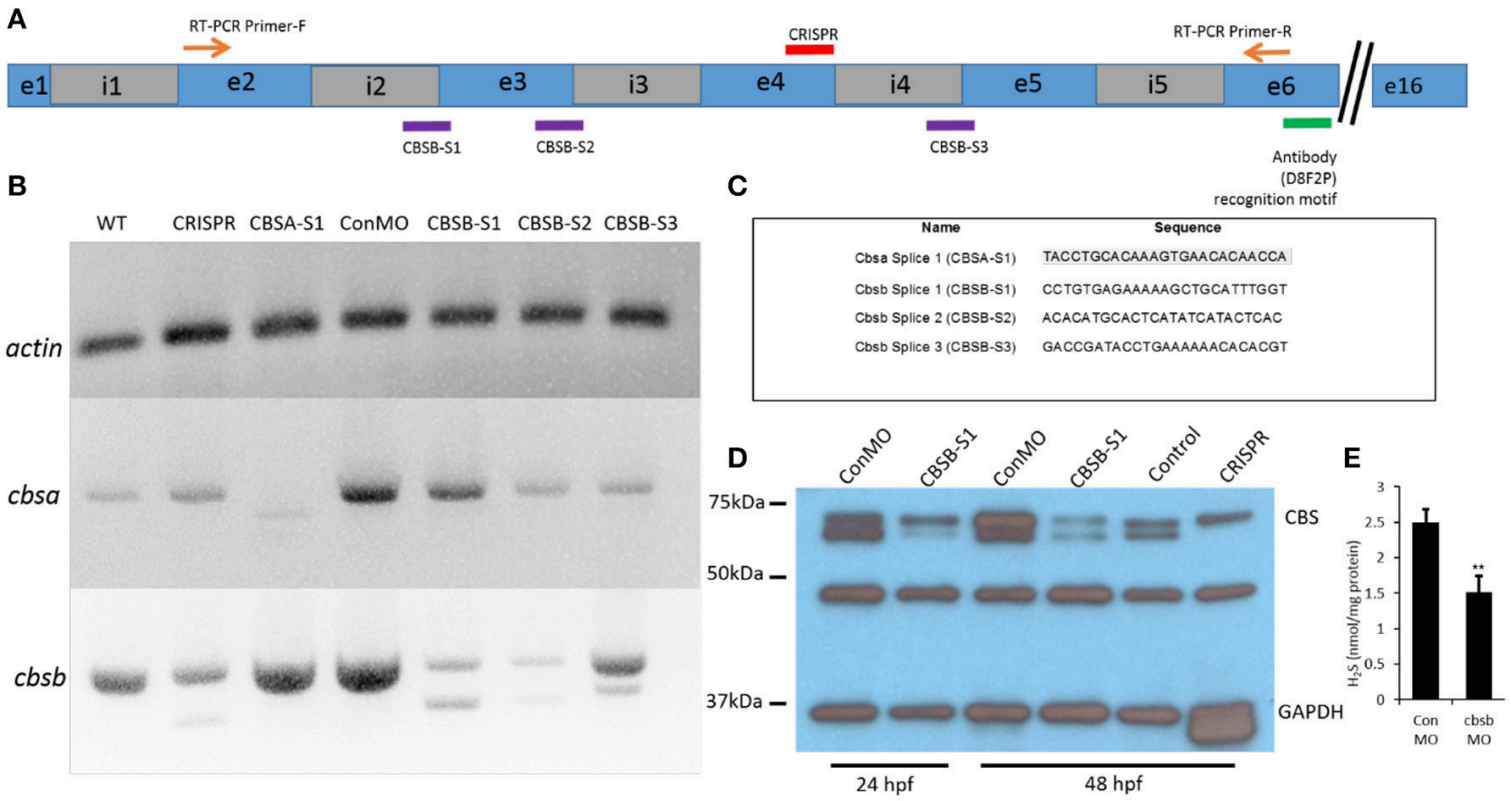
Loss-of-function efficacy studies. Panel **(A)** shows a “partial” cartoon representation of the *cbsb* genomic site with the location of the MO sites (purple rectangles) at appropriate intron (i) and exon (e) junctions, CRISPR-targeted site (red rectangle), site of RT-PCR forward (F) and reverse (R) primers, and the antibody recognition site. Panel **(B)** shows RT-PCR for three genes (*cbsb, cbsa, actin*) in total RNA from injected embryos (~24 hpf) (left to right): wild type (WT) control and *cbsb* CRISPR-injected fish, *cbsa* splice 1 (CBSA-S1), control morpholino (ConMO), *cbsb* splice1 (CBSB-S1), *cbsb* splice 2 (CBSB-S2), *cbsb* splice 3 (CBSB-S3). Panel **(C)** shows the sequence of the morpholinos used in this study. Panel **(D)** shows CBS and GAPDH western blots for ConMO, CBSB-S1 at 24 and 48 hpf along with control and *cbsb* CRISPR fish at 48 hpf. Panel **(E)** shows the comparison between CBSB-S1 MO and ConMO-injected embryos for hydrogen sulfide production. *n* = 3 for both groups (data from three experiments). Twenty embryos in each group in each experiment. ^**^P < 0.01.

The original article has been updated.

## Conflict of interest statement

The authors declare that the research was conducted in the absence of any commercial or financial relationships that could be construed as a potential conflict of interest.

