# Supplementary material for: Cystathionine β-Synthase Is Necessary for Axis Development in Vivo
Source: Front Cell Dev Biol. 2018 Feb 16;6:14. doi: 10.3389/fcell.2018.00014 (PMC5820354; doi:10.3389/fcell.2018.00014)
Supplement: Supplementary file 1 [file Image1.PDF]

Supplementary Material

Cystathionine β-synthase is necessary for axis development *in vivo*

Shubhangi Prabhudesai, Chris Koceja, Anindya Dey, Shahram Eisa-Beygi, Noah R. Leigh, Resham Bhattacharya*, Priyabrata Mukherjee*, & Ramani Ramchandran*.

***Correspondence:** Ramani Ramchandran: rramchan@mcw.edu, Resham Bhattacharya: Resham-Bhattacharya@OUHSC.edu, Priyabrata Mukherjee: Priyabrata-Mukherjee@OUHSC.edu

1 Supplementary Data – N/A.

2 Supplementary Figures and Tables

2.1 Supplementary Figures

See next page.

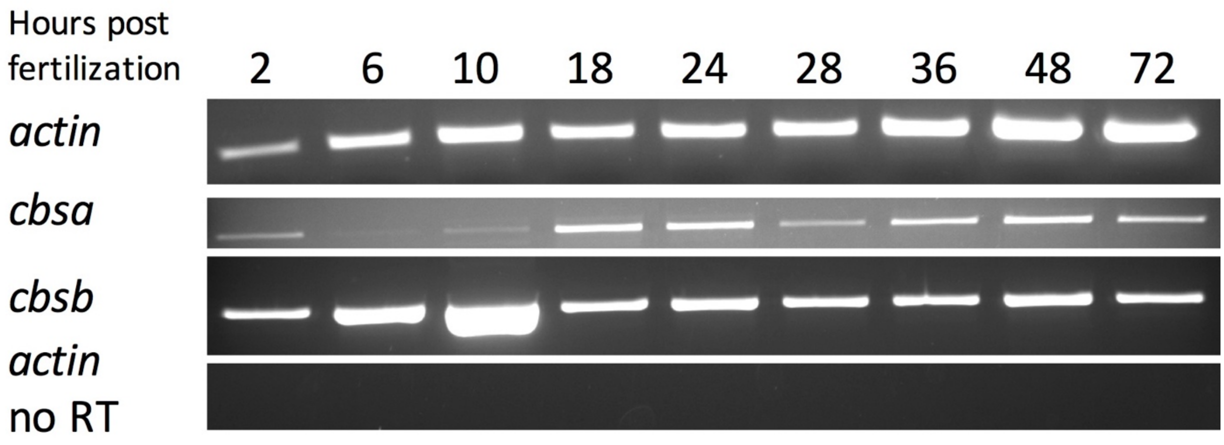

Supplementary Figure 1. Expression analysis of *cbsa* and *cbsb* genes in zebrafish embryos. qPCR using gene specific primers was performed on RNA isolated from select developmental stages as shown in panel.

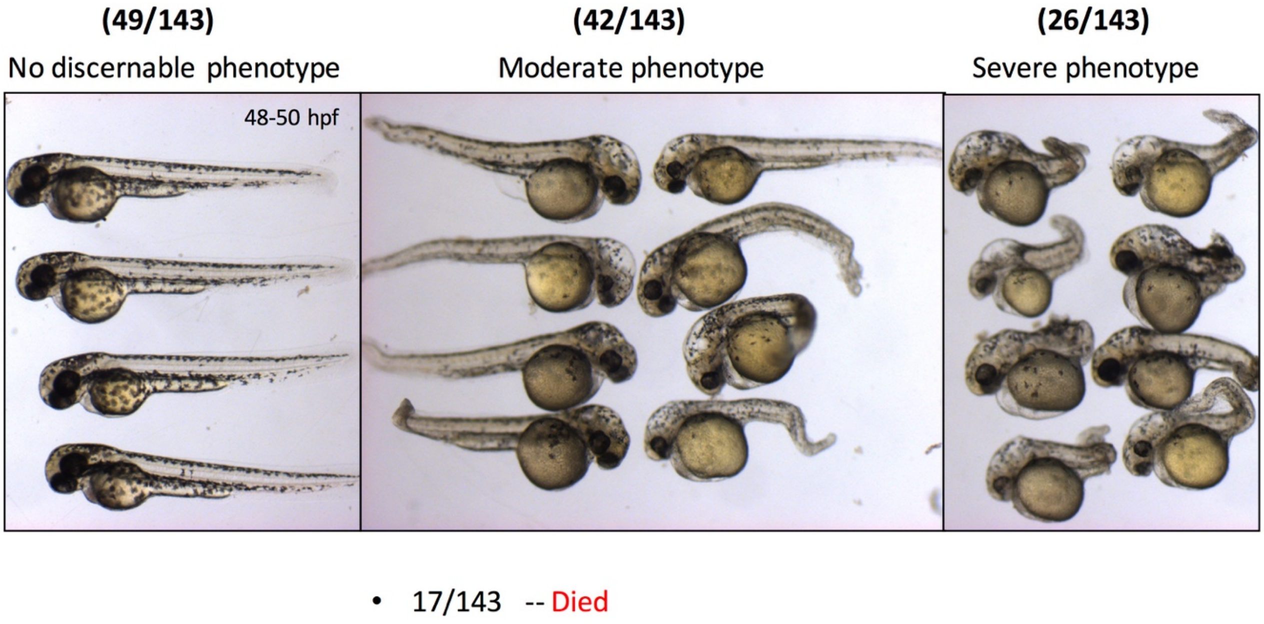

Supplementary Figure 2. Quantification of the *cbsb* morphant phenotype studies. Each panel shows the number of embryos that indicate the phenotype.

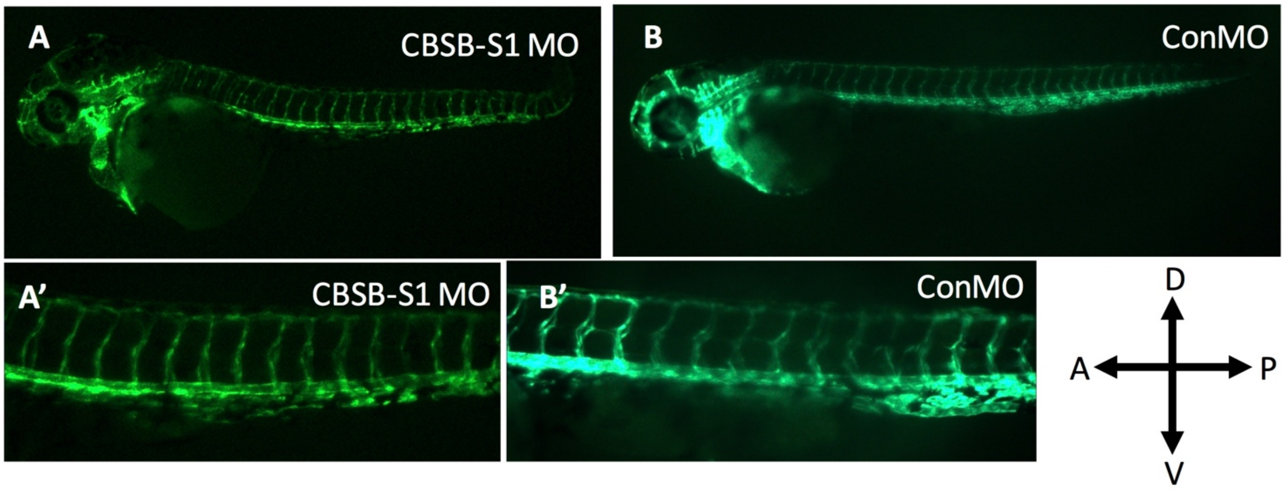

Supplementary Figure 3. Lack of vascular phenotype in *cbsb* (~50 hpf) morphants. The Tg (*flk*:EGFP) fish used in the figure 5 experiment carried a *VEGFR2* promoter (FLK) driving enhanced green fluorescent protein (EGFP) in the vasculature. A and A’ are *cbsb* splice MO1 (CBSB-S1) MO-injected fish while B and B’ are control (Con) MO-injected fish. A’ and B’ are high magnification images of the trunk vasculature. In the figure panels, the embryo orientation is left is anterior (A) and right is posterior (P) while top is dorsal (D) and bottom is ventral (V).

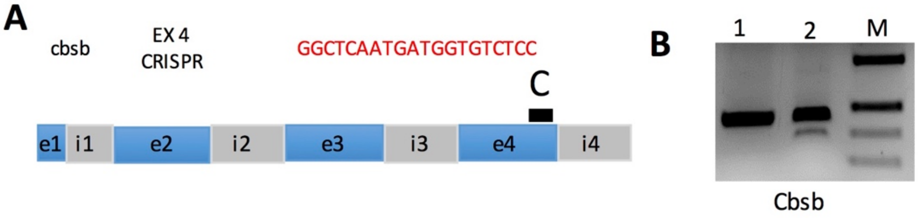

C

Sequence	% of F0 with genotype	Genotype
ACCCTTAAACCTGGAGACACCATCATTGAGCCTACATCAGGAA	42.2%	WT
ACCCTTAAACCTGGA**CACCATCATTGAGCCTACATCAGGAA	35.4%	2bp del
GCAAATATAAACTT*AGACACCATCATTGAGCCTACATCAGGAA	3.2%	1bp del and upstream point mut
CCAAATAAACCC***GACACCATCATTGAGCCTACATCAGGA	3.2%	3bp del and upstream point mut
ACCCTTAAACCTGG****ACCATCATTGAGCCTACATCAGGAA	3.2%	4bp del
ACCCTTAAACCT*GAG**ACCATCATTGAGCCTACATCAGGAA	3.2%	1 and 3bp del
ACCCTTAAACCT***GACACCATCATTGAGCCTACATCAGGAA	3.2%	3bp del
ACCCTTAAACCTG***ACACCATCATTGAGCCTACATCAGGAA	3.2%	3bp del
ACCCTTAAACCT*TA*A*ACCATCATTGAGCCTACATCAGGAA	3.2%	3 separate 1bp del and 1bp mut

Supplementary Figure 4. **CRISPR efficacy studies.** A shows a cartoon representation of the *cbsb* genomic site with the location of the CRISPR (C) (black rectangle) in exon 4 (e4). Sequence of CRISPR is provided in red font. B shows the RT-PCR of the targeted region in *cbsb* ex4 CRISPR template + *cbsb* sgRNA (lane1), *cbsb* ex4 CRISPR template + *cbsb* sgRNA + Cas9 Nuclease (lane 2), M-marker ladder. C shows the CRISPR genotyped fish in F0 generation, and the frequency of deletions (del) or upstream point mutations (mut).

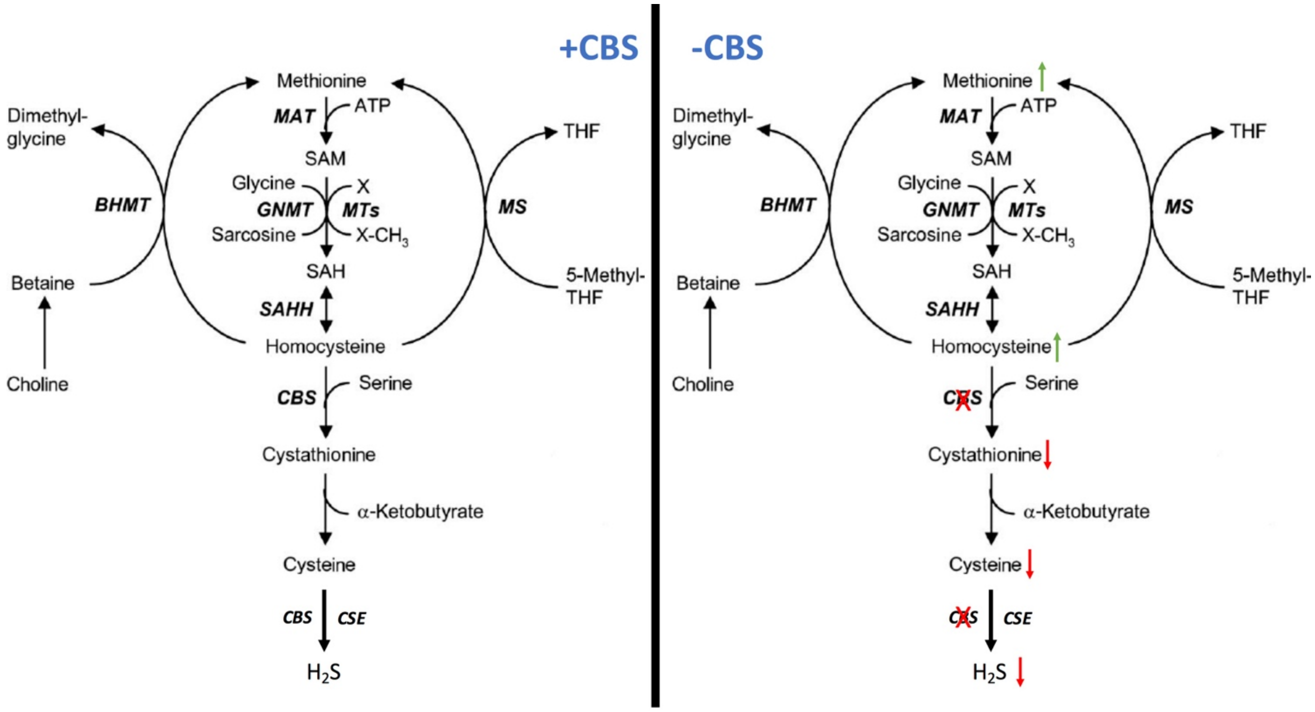

Supplementary Figure 5. Cysteine synthesis scheme. The scheme depicting the various steps involved in cysteine biosynthesis, and the metabolites involved in this pathway are indicated. The role of CBS enzyme in the conversion of homocysteine to cystathionine, the precursor for cysteine is shown. In addition, the role of betaine to convert homocysteine to methionine, which is one of the starting material for the cysteine synthesis process is shown. This pathway was modified from previous publication(Forestier et al., 2003).

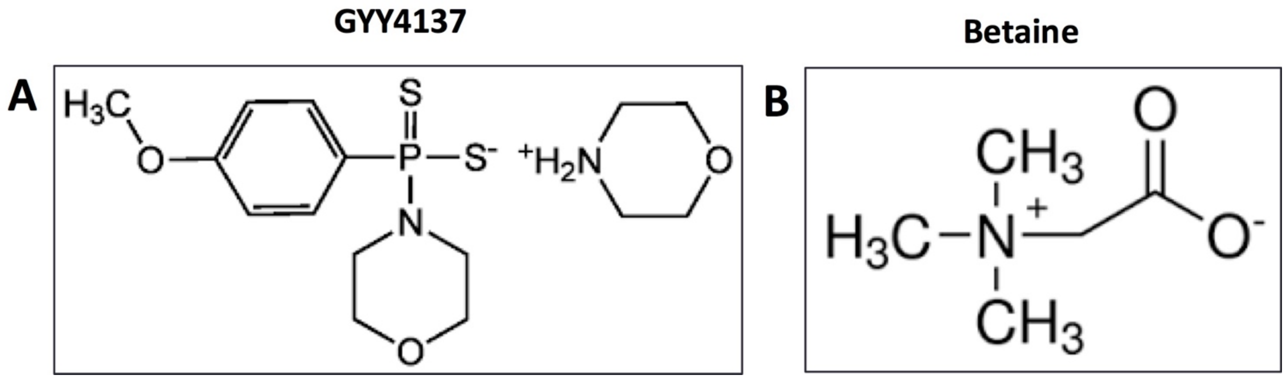

Supplementary Figure 6. Structures of small molecules used in this study. The structures of the molecules used in the rescue experiment is depicted.

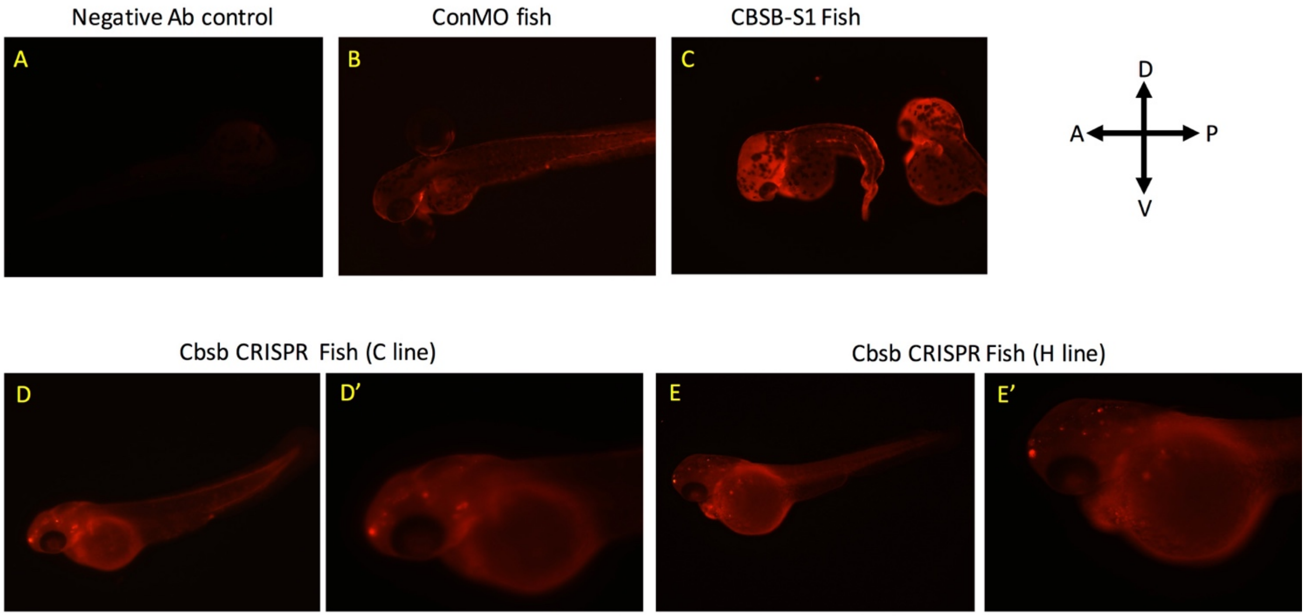

IF with homocysteine Ab at 48 hpf

Supplementary Figure 7. Homocysteine IF in *cbsb* morphant and CRISPR fish. IF with homocyteine antibody was performed in 48 hpf control MO (ConMO) fish (A) and *cbsb* splice1 MO (CBSB-S1) injected (C) fish. Panel A shows fish with no primary antibody control. D and E are *cbsb* CRISPR 48 hpf fish from C and H line respectively. D' and E' are high power images of the head and yolk region. Note the halo staining around the yolk, which is indicative of homocysteine proteins in yolk as previously reported (Matthews et al., 2009).

Supplementary References

Forestier, M., Banninger, R., Reichen, J., and Solioz, M. (2003). Betaine homocysteine methyltransferase: gene cloning and expression analysis in rat liver cirrhosis. *Biochim Biophys Acta* 1638**,** 29-34.

Matthews, R.P., Lorent, K., Manoral-Mobias, R., Huang, Y., Gong, W., Murray, I.V., Blair, I.A., and Pack, M. (2009). TNFalpha-dependent hepatic steatosis and liver degeneration caused by mutation of zebrafish S-adenosylhomocysteine hydrolase. *Development* 136**,** 865-875.
